# Antibiotic prescribing in people with and without diabetes: a population-based matched cohort study

**DOI:** 10.1093/jacamr/dlag074

**Published:** 2026-05-08

**Authors:** Francesco Dernie, Iain M Carey, Liza Bowen, Umar A R Chaudhry, Selma Audi, Emma Baker, Derek G Cook, Tess Harris, Julia A Critchley

**Affiliations:** School of Health & Medical Sciences, City St George’s, University of London, London SW17 0RE, UK; School of Health & Medical Sciences, City St George’s, University of London, London SW17 0RE, UK; School of Health & Medical Sciences, City St George’s, University of London, London SW17 0RE, UK; School of Health & Medical Sciences, City St George’s, University of London, London SW17 0RE, UK; School of Health & Medical Sciences, City St George’s, University of London, London SW17 0RE, UK; School of Health & Medical Sciences, City St George’s, University of London, London SW17 0RE, UK; School of Health & Medical Sciences, City St George’s, University of London, London SW17 0RE, UK; School of Health & Medical Sciences, City St George’s, University of London, London SW17 0RE, UK; School of Health & Medical Sciences, City St George’s, University of London, London SW17 0RE, UK

## Abstract

**Aims:**

To characterize utilization of antibiotics, including those with higher antimicrobial resistance potential, among people with type 1 diabetes and type 2 diabetes in England.

**Methods:**

Cohort study in the Clinical Practice Research Datalink, including 524 285 patients with type 2 diabetes and 33 843 with type 1 diabetes alive on 1 January 2015, matched to two non-diabetes patients on age-sex-practice and followed up to 31 December 2019. Antibiotic counts and rates were calculated for UK AWaRe (Access, Watch, Reserve) antibiotic categories. Poisson regression estimated rate ratios (RRs) of antibiotic prescriptions for diabetes versus non-diabetes, adjusting for confounders including infection count, and further stratified by age, sex, ethnicity, and deprivation.

**Results:**

Higher antibiotic prescription rates were found in diabetes groups (type 1 diabetes RR = 2.21, type 2 diabetes RR = 1.57), which were only partially attenuated after full adjustment (type 1 diabetes RR = 1.56, 95% CI 1.55–1.57, type 2 diabetes RR = 1.14, 95% CI 1.13–1.14). Elevated estimates were seen across both Access and Watch AWaRe categories. Prescription rates were higher in females and White participants, and increased with increasing age bands and deprivation.

**Conclusions:**

People with diabetes are exposed to more courses of antibiotics even when controlling for infection count, including broader spectrum antibiotics which require monitoring and risk driving antimicrobial resistance.

## Introduction

Type 2 diabetes mellitus (T2DM) affects around 529 million people worldwide, with a further 8.4 million individuals living with type 1 diabetes mellitus (T1DM).^1,2^ The prevalence of both diseases is predicted to rise in the coming decades, with an estimated 1.3 billion cases of T2DM by 2050, and 17.4 million cases of T1DM by 2040.^1,2^ Alongside well-established complications including cardiovascular disease, renal impairment, and neuropathy, there is increasing recognition of the greater susceptibility to, and poorer outcomes from, infections among people with diabetes.^3,4^

Large cohort studies using primary care data in the United Kingdom (UK) have previously shown that people with T2DM or T1DM had a higher rate of infections, infection-related hospitalizations, and infection-related mortality, compared with people without diabetes.^5–8^ This increased infection risk would be expected to lead to higher antibiotic use among people with diabetes, but real-world evidence of this is in the UK is lacking.

Identifying groups with increased exposure to antibiotics, especially broader-spectrum antibiotics reserved for more severe infections, is of key public health interest given the strong link between antibiotic consumption and antimicrobial resistance (AMR), which was associated with an estimated 4.7 million deaths globally in 2021.^9,10^ Repeated antibiotic exposure has been associated with an increased risk of AMR at the individual patient level, particularly in the context of primary care prescribing.^11^ The UK government's 2024–2029 national action plan for confronting AMR identifies a critical need for understanding antimicrobial use in high-risk populations and identifying the socioeconomic and clinical factors which are associated with this.^12^

In this study, we utilize a large primary care database in England to comprehensively describe and quantify antibiotic prescribing among people with T1DM and T2DM, exploring overall antibiotic course counts, the duration of acute prescriptions, and specific antibiotic classes of national and global public health relevance. We consider the impact of adjusting for confounding factors on antibiotic utilization, including underlying infection count, and report differences compared with non-diabetic patients based on key sociodemographic factors including age, sex, ethnicity, and deprivation.

## Methods

### Data source

The Clinical Practice Research Datalink (CPRD) Aurum database is a UK primary care database jointly sponsored by the Medicines and Healthcare products Regulatory Agency (MHRA) and the National Institute for Health and Care Research (NICE).^13^ It contains pseudonymized longitudinal records for patients registered with general practices using EMIS Web software, covering over 16 million active patients from 1784 practices in England as of 2024.^14^ Over 90% of practices provide linkage to external datasets,^15^ including the index of multiple deprivation (IMD), a small-area measure of socioeconomic deprivation. Ethical approval was granted by CPRD's Research Data Governance (protocol number 21_000592).

### Study design and participants

We conducted a matched cohort study including all patients aged 18–90 active in CPRD on 1 January 2015 with ≥1 year of registration. Initially 8 722 348 patients were eligible for inclusion (Figure S1, available as Supplementary data at *JAC-AMR* Online). Diabetes status (T1DM, T2DM, or type unknown) was classified using a previously described algorithm incorporating diagnostic Read codes and anti-diabetic medications (Table S1, Figure S2).^6^

Each person with T1DM or T2DM was matched to two patients without diabetes on age, sex and practice (*n* = 1 097 364). Practice matching was chosen to reduce the effect of inter-practice variation in prescribing patterns. Over 99.5% of T1DM/T2DM patients were matched. A total of 524 299 patients with T2DM, and 33 844 patients with T1DM were initially selected. Patients with zero days of follow-up time were excluded, leaving 524 285 patients with T2DM, and 33 843 patients with T1DM.

Follow-up continued to the earliest of patient death or deregistration, practice leaving the CPRD, or 31 December 2019. Follow-up was designed to end just prior to the start of the COVID-19 pandemic, which caused significant changes in infection event coding, antibiotic prescription patterns, and disease transmission.^16,17^

### Antibiotic classification and prescription counts

Antibiotics prescribed during follow-up were identified and grouped into 31 classes (Table S1-S2), and assigned an ‘Access, Watch, and Reserve’ (AWaRe) category. AWaRe is an antibiotic classification system developed originally by the WHO in 2017 as a tool to support global antibiotic stewardship efforts.^18^ Access antibiotics are those with a narrow spectrum, fewer side effects, lower costs, and lower resistance potential, and are first or second choice antibiotics for most common infections. Watch antibiotics are broader spectrum with higher resistance potential and are thus indicated for a more limited number of infections with careful monitoring. Reserve antibiotics are ‘last resort’ antibiotics used for highly selected patients and are closely monitored. In 2019, the WHO categorization was adapted for England, with resulting changes in individual antibiotic classification based on local resistance patterns, adverse drug events, and national antimicrobial stewardship objectives.^19^ Given the setting of this study, we used the England AWaRe classification for our analyses (Table S2). Of note, the AWaRe classification is by specific antibiotic, so antibiotic classes may have members in different AWaRe categories.

The total number of antibiotic courses were counted for each patient, summarized by diabetes group, and further stratified by class and AWaRe status. There were no limits placed on the number of antibiotic prescriptions an individual could receive within the study, to ensure the full spectrum of antibiotic exposure was captured in our data.

To explore the effect of long-term antibiotics on overall antibiotic exposure between groups, we performed a sub-group analysis of ‘high prescription level’ individuals, who were defined as having a prescription rate equivalent to fortnightly (or more frequently) during at least 6 months of follow-up. We calculated the proportion of all antibiotic prescriptions in the study cohort these high-prescription individuals accounted for. The dominant antibiotic (accounting for the most courses) for each individual in the high prescription group was identified and compared.

### Infection outcomes

Each infection Read code was classified based on the broad causative infectious agent: ‘bacterial’, ‘viral’, ‘fungal’, ‘mycobacterial’, or ‘other’ (parasitic or helminths). Non-specific Read codes (such as ‘skin rash’), which may have an underlying bacterial aetiology were classified as ‘possibly bacterial’. In line with our previous analyses, repeated infection Read codes for the same patient within 90 days were assumed to be the same infection event and not counted.^7^ For our primary analyses, infection count was defined as the combined counts of bacterial and possibly bacterial infections, as these were those most likely to be treated with antibiotics.

### Antibiotic duration for acute infections

We analysed the duration of antibiotic courses for three acute infections: urinary tract infections (UTI), cellulitis, and community-acquired pneumonia (CAP). These were chosen as they are common and important acute infections which generally present to primary care services and have clear guidelines for treatment. Read codes for these infections were identified from a list of all infection Read codes recorded during the study period (Table S3). We then extracted the total quantity, and duration of treatment (in days) for selected antibiotics prescribed within 7 days from infection event date. We chose the first 7 days to ensure a tight temporal link between the infection code and the antibiotic prescription, in order to increase the likelihood that the antibiotic was prescribed acutely for the infection in question. The antibiotics chosen for comparison were those indicated for the treatment of these conditions by the National Institute for Health and Care Excellence (NICE) Clinical Knowledge Summaries (Table S4).^20–23^ Where the duration of an antibiotic was not recorded, it was calculated using the recorded total quantity prescribed, divided by the guideline-recommended daily dosing regimen, divided by the relative strength of the prescribed drug (e.g. where 1 is the standard dose, and 2 would be half-strength tablets of which two were taken for the same dose). Prescriptions identified as repeat medications, and courses lasting longer than 14 days, were excluded as these were likely related to long-term prophylaxis rather than acute events. The median course duration and interquartile range was calculated for each individual antibiotic. Findings were split by sex if guidelines recommended different lengths of treatment for males and females.

### Other covariates

We extracted data on smoking history, BMI, co-morbidities, and IMD as of 1 January 2015, and ethnicity codes from anytime on the patient's record. Patients were grouped into four broad self-defined ethnicity categories (White, South Asian, Black, Mixed/Other) based on recorded Read codes.^7^ We were able to classify ethnicity for approximately 90% of patients with T1DM and T2DM. IMD is classified into five quintiles with 1 being the least deprived and 5 the most. We selected 12 chronic conditions for comorbidity counts that were routinely collected as part of the Quality and Outcomes Framework (QOF), a UK-wide system for performance management and payment of GPs in primary care.^24^ These were atrial fibrillation, cancer, chronic obstructive pulmonary disease, coronary heart disease, chronic kidney disease, dementia, epilepsy, heart failure, hypertension, peripheral vascular disease, serious mental illness, and stroke.

### Statistical methods

Raw rates of antibiotic prescriptions per 1000 person-years of follow-up were calculated for all antibiotics, by antibiotic class, and by AWaRe status. Poisson regression, with a log link, conditioned for match-sets (matched on age, sex, and GP practice), was used to model the count of antibiotic prescriptions as the dependent variable and diabetes status (T1DM or T2DM versus non-diabetes) as the primary exposure. An offset term of the log of the total years of follow-up was used to account for varying follow-up time in the study. Models were initially fitted without further adjustment. For all antibiotics and the three individual AWaRe groups, we then ran models adjusted for (1) BMI, IMD, ethnicity, smoking status and comorbidity count and (2) the same variables and additionally infection count. Rate ratios (RRs) and corresponding 95% CIs were calculated using Wald-based standard errors.

To investigate whether the association between diabetes status and antibiotic prescribing varied by sociodemographic characteristics (age, sex, IMD quintile, ethnicity), we ran further Poisson regression models with interaction terms, adjusting for the same potential confounders as our main analyses. Stratum-specific RRs and 95% CIs were derived by combining the main diabetes effects with interaction coefficients and their variances. Too few Reserve antibiotic prescriptions were present for stratified analyses.

### Software

R version 4.4.2 was used for all data handling, statistical analyses, and data visualization. The ‘gnm’ package was used for Poisson modelling, with elimination of match identifiers to condition for age, sex, and GP practice. All plots were generated using ggplot2. The analytical code used for this study can be accessed via https://github.com/francescodernie/diabetes_antibiotics.

## Results

### Population characteristics

People with T1DM were on average ∼20 years younger than those with T2DM (45.6 versus 66.2 years, Table 1). In both cohorts, the White ethnic group was older (Figure S3A and B). The sex ratio was similar (56% male in T2DM, 58% in T1DM). People with T2DM had higher recorded BMI and comorbidity counts. A slightly higher proportion of the T2DM group were from the more deprived quintiles. Ethnicity was better recorded in the diabetes groups, with higher proportions of non-white ethnicities in the T2DM group (20.2%) compared to T1DM (7.5%). Mean follow-up was 4.3 years.

**Table 1.** Baseline characteristics of patients with type 1 (T1DM) and type 2 (T2DM) diabetes, and matched non-diabetic patients, on 1 January 2015

	T1DM		T2DM	
Baseline characteristic	Diabetes	Non-diabetes	Diabetes	Non-diabetes
	(*n* = 33 843)	(*n* = 67 587)	(*n* = 524 285)	(*n* = 1 029 722)
Sex				
Female	14 153 (41.8%)	28 275 (41.8%)	231 635 (44.2%)	455 456 (44.2%)
Male	19 690 (58.2%)	39 312 (58.2%)	292 650 (55.8%)	574 266 (55.8%)
Age (years)				
Mean (SD)	45.6 (16.1)	45.5 (16.1)	66.2 (13.0)	66.1 (13.0)
Index of multiple deprivation				
1 (least deprived)	7066 (20.9%)	14 737 (21.8%)	87 405 (16.7%)	203 816 (19.8%)
2	7058 (20.9%)	14 240 (21.1%)	97 409 (18.6%)	210 349 (20.4%)
3	6704 (19.8%)	13 100 (19.4%)	101 492 (19.4%)	201 853 (19.6%)
4	6547 (19.3%)	13 067 (19.3%)	116 192 (22.2%)	209 973 (20.4%)
5 (most deprived)	6439 (19.0%)	12 390 (18.3%)	121 410 (23.2%)	202 977 (19.7%)
Missing	29 (0.1%)	53 (0.1%)	377 (0.1%)	754 (0.1%)
Ethnicity				
Black	594 (1.8%)	1524 (2.3%)	22 248 (4.2%)	29 583 (2.9%)
Mixed/Other	1094 (3.2%)	2816 (4.2%)	30 076 (5.7%)	37 383 (3.6%)
South Asian	846 (2.5%)	2644 (3.9%)	53 776 (10.3%)	46 091 (4.5%)
White	27 319 (80.7%)	46 686 (69.1%)	367 252 (70.0%)	760 459 (73.9%)
Missing	3990 (11.8%)	13 917 (20.6%)	50 933 (9.7%)	156 206 (15.2%)
BMI				
<25	13 727 (40.6%)	24 639 (36.5%)	81 021 (15.5%)	346 358 (33.6%)
25–30	12 302 (36.3%)	19 485 (28.8%)	175 457 (33.5%)	380 885 (37.0%)
30–40	6846 (20.2%)	10 861 (16.1%)	218 665 (41.7%)	205 905 (20.0%)
>40	650 (1.9%)	1387 (2.1%)	46 122 (8.8%)	19 458 (1.9%)
Missing	318 (0.9%)	11 215 (16.6%)	3020 (0.6%)	77 116 (7.5%)
Smoking status				
Current smoker	6948 (20.5%)	14 239 (21.1%)	74 636 (14.2%)	160 633 (15.6%)
Ex-smoker	11 403 (33.7%)	18 787 (27.8%)	252 485 (48.2%)	416 795 (40.5%)
Never smoker	15 401 (45.5%)	32 033 (47.4%)	196 605 (37.5%)	436 148 (42.4%)
Missing	91 (0.3%)	2528 (3.7%)	559 (0.1%)	16 146 (1.6%)
Comorbidities				
Coronary heart disease	1995 (5.9%)	1485 (2.2%)	92 799 (17.7%)	85 352 (8.3%)
Chronic kidney disease	2741 (8.1%)	1291 (1.9%)	94 825 (18.1%)	92 608 (9.0%)
Heart failure	448 (1.3%)	259 (0.4%)	23 423 (4.5%)	19 241 (1.9%)
Hypertension	9278 (27.4%)	7847 (11.6%)	329 898 (62.9%)	349 629 (34.0%)
Peripheral arterial disease	1076 (3.2%)	266 (0.4%)	22 563 (4.3%)	16 629 (1.6%)
Atrial fibrillation	492 (1.5%)	807 (1.2%)	39 834 (7.6%)	53 060 (5.2%)
Cancer	879 (2.6%)	1728 (2.6%)	40 926 (7.8%)	74 040 (7.2%)
Epilepsy	822 (2.4%)	963 (1.4%)	8483 (1.6%)	16 732 (1.6%)
Serious mental health condition	332 (1.0%)	588 (0.9%)	11 314 (2.2%)	11 297 (1.1%)
Dementia	158 (0.5%)	190 (0.3%)	12 451 (2.4%)	20 505 (2.0%)
Chronic obstructive pulmonary disease	520 (1.5%)	1004 (1.5%)	33 211 (6.3%)	55 793 (5.4%)
Stroke/TIA	978 (2.9%)	759 (1.1%)	39 507 (7.5%)	47 510 (4.6%)

### Antibiotic prescription rates

During follow-up, 74.1% of patients with T2DM (*n* = 388 449) were prescribed at least one antibiotic, compared with 62.4% of matched non-diabetes patients (*n* = 642 274) (Table 2). The disparity was slightly larger in the T1DM analysis (68.2%, *n* = 23 089 versus 51.2%, *n* = 34 631). Diabetes groups also had higher proportions of subjects with at least one prescription in each of the AWaRe categories (Table 2).

**Table 2.** Summary of antibiotic prescription rates and rate ratios during 2015–2019 and stratified by AWaRe status, among patients with diabetes versus matched non-diabetes patients

	T1DM (*n* = 33 843)			Non-diabetes (*n* = 67 587)			Rate ratios		
	Subjects (%)^a^	Courses	Rate^b^	Subjects (%)^a^	Courses	Rate^b^	Unadjusted^c^	Adjusted ^d^	Adjusted ^e^
All antibiotics	23 089 (68.2)	160 081	1120.5	34 631 (51.2)	151 238	518.6	2.21 (2.19–2.22)	1.84 (1.83–1.86)	1.56 (1.55–1.57)
Access	21 163 (62.5)	106 146	743.0	31 088 (46.0)	109 525	375.6	2.02 (2.00–2.04)	1.70 (1.68–1.72)	1.43 (1.42–1.45)
Watch	12 055 (35.6)	54 162	379.1	14 965 (22.1)	42 948	147.3	2.62 (2.59–2.65)	2.15 (2.12–2.18)	1.84 (1.81–1.87)
Reserve	90 (0.3)	794	5.6	15 (0.02)	81	0.3	22.17 (17.49–28.11)	24.52 (13.84–43.44)	23.78 (13.46–42.01)

	T2DM (*n* = 524 285)			Non-diabetes (*n* = 1 029 722)			Rate ratios		
	Subjects (%)^a^	Courses	Rate^b^	Subjects (%)^a^	Courses	Rate^b^	Unadjusted^c^	Adjusted ^d^	Adjusted ^e^
All antibiotics	388 449 (74.1)	2 918 090	1316.5	642 274 (62.4)	3 805 948	853.5	1.57 (1.57–1.57)	1.22 (1.22–1.22)	1.14 (1.13–1.14)
Access	363 547 (69.3)	2 098 960	946.9	590 346 (57.3)	2 764 199	619.9	1.55 (1.55–1.56)	1.21 (1.21–1.21)	1.13 (1.13–1.13)
Watch	203 263 (38.8)	854 098	385.3	305 665 (29.7)	1 082 435	242.7	1.63 (1.62–1.63)	1.25 (1.25–1.25)	1.16 (1.16–1.17)
Reserve	443 (0.08)	3529	1.6	540 (0.05)	6356	1.4	1.10 (1.05–1.15)	0.91 (0.86–0.96)	0.86 (0.81–0.91)

^a^Number (%) of each group with at least one prescription of an antibiotic during the study follow-up period.

^b^Per 1000 person-years.

^c^Conditioned on match sets (age, sex, and GP practice matched).

^d^Adjusted for BMI, ethnicity, IMD, smoking status, and comorbidity count.

^e^Adjusted for BMI, ethnicity, IMD, smoking status, comorbidity count, and infection count (bacterial + possibly bacterial infections).

Table 2 summarizes antibiotic prescription rates for people with and without diabetes, and resulting RRs. Prescription rates for all antibiotics were higher in the T2DM cohort compared to the T1DM cohort (1316 versus 1120 per 1000pys).

Patients with diabetes had higher antibiotic prescription rates compared to people without diabetes (T1DM: RR 2.21, 95% CI 2.19–2.22; T2DM: RR 1.57, 95% CI 1.57–1.57). The difference in RRs between the two diabetes groups is due to the relatively older T2DM cohorts, where the underlying prescription rate in the matched non-diabetes patients is higher due to age-related infection risk. A sensitivity analysis of younger (<50 years) T2DM patients shows a lower prescription rate than the overall T2DM cohort (956 per 1000pys), but a RR similar to the T1DM cohort (RR 2.25, 95% CI 2.24–2.27) (Table S5).

RRs were partially attenuated by increasing adjustment including underlying infection count (T1DM RR 1.56, 95% CI 1.55–1.57; T2DM RR 1.14, 95% CI 1.13–1.14). Following full adjustment, both T1DM and T2DM groups had higher RRs for both Access (T1DM RR 1.43, 95% CI 1.42–1.45; T2DM RR 1.13, 95% CI 1.13–1.13) and Watch (T1DM 1.84, RR 1.81–1.87; T2DM RR 1.16, 95% CI 1.16–1.17) antibiotics. During the study period, 72% of antibiotic courses prescribed to patients with T2DM were from the Access class (and 73% for matched non-diabetes patients), compared with 66% of antibiotics prescribed to patients with T1DM (and 72% of matched non-diabetes patients). Course counts and RRs for individual antibiotic classes are available in Table S6.

Fewer than 0.06% of patients (*n* = 1088) were prescribed a Reserve antibiotic overall during the study period in primary care (Table 2). The resulting fully adjusted RR for T1DM was 23.78 (95% CI 13.46–42.01), whereas for T2DM, this was 0.86 (95% CI 0.81–0.91) (Table 2). Ninety-four percent of Reserve prescriptions were for inhaled colistin, which is used long-term in the treatment of respiratory pseudomonal infections in people with chronic lung conditions such as cystic fibrosis or bronchiectasis (Table S7).

### Stratification of results by age, sex, ethnicity, and deprivation

Antibiotic prescription rates were further stratified in both diabetes groups by age, sex, ethnic group, and IMD quintile. Within each stratum, RRs were calculated for all antibiotics and the Access and Watch categories of antibiotics.

Prescription rates for both diabetes cohorts were higher compared to matched non-diabetic cohorts within each stratum (Tables 3 and 4). Female patients had between 60% and 90% higher prescription rates compared with males. Prescription rates increased gradually with increasing deprivation. Prescription rates also increased with increasing age bands, while the resulting RRs decreased due to the underlying rate of antibiotic use among the matched non-diabetic patients increasing to a greater extent with age (Tables 3 and 4). In the T1DM cohort, people from non-White ethnic groups had lower prescription rates overall compared to the White ethnic group, with the South Asian ethnic group having the highest RR between people with and without diabetes (1.82, 95% CI 1.71–1.94) (Table 3). In the T2DM cohort, non-White ethnic groups again had lower prescription rates compared to people of White ethnicity, but slightly higher RRs when comparing people with and without diabetes in each group (RRs by ethnicity: White 1.11 versus Black 1.26, South Asian 1.20, Mixed/Other 1.24) (Table 4). The higher prescription rates in White groups were not readily explained by their higher mean ages, as prescription rate differences were maintained across age groups (Figures S4 and S5).

**Table 3.** Rates per 1000 person years of all antibiotics, Access antibiotics, and Watch antibiotics, for people with type 1 diabetes (T1DM), stratified by sex, age, ethnicity, and deprivation

	All antibiotics			Access			Watch		
Strata	T1DM	Controls	Rate ratio	T1DM	Controls	Rate ratio	T1DM	Controls	Rate ratio
Female	1502.9	717.0	1.51 (1.49–1.53)	1012.1	531.1	1.38 (1.36–1.40)	495.4	191.4	1.80 (1.77–1.84)
Male	846.7	377.4	1.63 (1.61–1.65)	550.3	264.9	1.49 (1.46–1.51)	295.8	115.9	1.84 (1.80–1.88)
18–30	996.7	403.6	1.78 (1.75–1.82)	623.0	283.8	1.51 (1.47–1.56)	362.1	123.5	2.19 (2.12–2.27)
31–40	1010.0	394.2	1.97 (1.92–2.01)	645.2	283.2	1.69 (1.64–1.73)	367.6	113.1	2.56 (2.47–2.66)
41–50	962.9	411.0	1.66 (1.62–1.69)	631.6	295.7	1.50 (1.47–1.54)	337.0	118.7	1.94 (1.88–2.00)
51–60	1091.2	502.1	1.55 (1.52–1.58)	735.4	356.7	1.52 (1.48–1.55)	359.8	151.1	1.59 (1.54–1.64)
61–70	1366.1	699.6	1.27 (1.24–1.29)	932.9	507.9	1.20 (1.17–1.23)	433.9	194.3	1.40 (1.35–1.45)
71–80	1801.5	1024.5	1.28 (1.25–1.31)	1303.9	771.6	1.23 (1.19–1.26)	503.9	262.4	1.38 (1.32–1.44)
81+	1970.4	1350.4	1.11 (1.06–1.16)	1342.2	1072.0	0.98 (0.93–1.03)	666.7	293.1	1.54 (1.42–1.67)
White	1129.7	575.7	1.51 (1.49–1.52)	749.3	417.1	1.37 (1.36–1.39)	381.0	163.1	1.76 (1.73–1.79)
Black	823.8	398.9	1.47 (1.36–1.60)	609.8	287.2	1.38 (1.25–1.53)	226.6	114.7	1.62 (1.40–1.88)
South Asian	1052.8	432.5	1.82 (1.71–1.94)	715.8	310.2	1.78 (1.64–1.92)	342.5	126.1	1.76 (1.59–1.96)
Mixed/Other	917.6	361.2	1.59 (1.50–1.69)	620.2	266.1	1.53 (1.42–1.65)	317.8	97.8	1.73 (1.55–1.92)
IMD 1 (least deprived)	995.8	480.2	1.45 (1.42–1.48)	656.2	337.3	1.34 (1.31–1.38)	338.8	145.1	1.66 (1.60–1.72)
IMD 2	1086.1	504.2	1.61 (1.58–1.65)	717.0	367.3	1.48 (1.44–1.52)	381.6	141.3	1.90 (1.83–1.97)
IMD 3	1120.4	523.0	1.50 (1.47–1.54)	733.9	381.1	1.40 (1.37–1.44)	386.1	146.3	1.69 (1.63–1.76)
IMD 4	1127.8	518.1	1.60 (1.57–1.64)	764.2	376.8	1.47 (1.43–1.51)	362.3	146.5	1.80 (1.74–1.88)
IMD 5 (most deprived)	1289.2	579.1	1.62 (1.59–1.65)	857.1	425.3	1.41 (1.38–1.45)	429.0	159.1	2.07 (2.00–2.15)

Rate ratios (95% CI) are fully adjusted for all covariates.

**Table 4.** Rates per 1000 person years of all antibiotics, Access antibiotics, and Watch antibiotics, for people with type 2 diabetes (T2DM), stratified by sex, age, ethnicity, and deprivation

	All antibiotics			Access			Watch		
Strata	T2DM	Controls	Rate ratio	T2DM	Controls	Rate ratio	T2DM	Controls	Rate ratio
Female	1610.4	1057.8	1.11 (1.11–1.11)	1168.8	779.0	1.10 (1.09–1.10)	461.2	290.6	1.15 (1.15–1.16)
Male	1082.7	691.0	1.17 (1.16–1.17)	770.5	493.3	1.16 (1.16–1.17)	325.0	204.7	1.18 (1.17–1.18)
18–30	1204.7	456.3	1.92 (1.85–1.98)	769.4	333.6	1.61 (1.55–1.68)	432.3	128.8	2.64 (2.48–2.80)
31–40	979.6	424.6	1.52 (1.50–1.55)	677.4	309.9	1.45 (1.43–1.48)	310.9	118.8	1.70 (1.65–1.75)
41–50	940.5	424.7	1.50 (1.49–1.51)	664.9	307.7	1.46 (1.45–1.48)	285.4	121.0	1.59 (1.57–1.62)
51–60	1028.5	513.5	1.37 (1.37–1.38)	728.5	370.4	1.35 (1.34–1.36)	309.7	147.9	1.43 (1.42–1.44)
61–70	1219.2	748.4	1.16 (1.16–1.17)	870.6	537.0	1.16 (1.15–1.16)	362.3	218.7	1.18 (1.17–1.19)
71–80	1501.3	1074.8	1.05 (1.05–1.06)	1084.2	780.3	1.05 (1.05–1.05)	435.8	304.8	1.07 (1.07–1.08)
81+	1859.6	1456.7	1.02 (1.02–1.02)	1372.2	1074.6	1.02 (1.02–1.03)	515.8	402.4	1.02 (1.01–1.03)
White	1411.8	917.5	1.11 (1.11–1.11)	1012.7	666.2	1.10 (1.10–1.10)	416.2	261.2	1.14 (1.14–1.15)
Black	731.7	465.1	1.26 (1.24–1.27)	551.1	348.8	1.25 (1.23–1.27)	189.8	121.8	1.26 (1.22–1.29)
South Asian	1073.7	674.4	1.20 (1.19–1.21)	790.4	491.4	1.22 (1.21–1.24)	294.4	188.9	1.12 (1.10–1.14)
Mixed/Other	920.9	573.4	1.24 (1.23–1.25)	670.9	415.8	1.25 (1.24–1.27)	259.4	164.5	1.19 (1.17–1.22)
IMD 1 (least deprived)	1208.6	788.4	1.13 (1.12–1.13)	861.3	564.4	1.13 (1.12–1.14)	363.7	233.2	1.12 (1.11–1.13)
IMD 2	1264.5	823.1	1.13 (1.12–1.13)	904.5	595.0	1.11 (1.10–1.11)	378.2	238.0	1.18 (1.17–1.19)
IMD 3	1311.4	832.1	1.16 (1.15–1.16)	938.7	603.2	1.15 (1.15–1.16)	389.0	238.3	1.18 (1.17–1.19)
IMD 4	1319.9	864.3	1.15 (1.15–1.16)	947.3	632.0	1.14 (1.13–1.15)	387.5	240.7	1.19 (1.18–1.20)
IMD 5 (most deprived)	1438.3	963.6	1.12 (1.11–1.12)	1050.2	707.8	1.11 (1.10–1.12)	401.9	264.4	1.14 (1.13–1.15)

Rate ratios (95% CI) are fully adjusted for all covariates.

### Subgroup analysis of participants with high levels of prescriptions

We identified 2141 patients who met the criteria for our ‘high prescription’ cohort (Table S8). A greater proportion of patients with diabetes received high rates of antibiotics compared to patients without diabetes (T1DM 0.14% versus 0.02%, and T2DM 0.22% versus 0.09%). The median number of antibiotic courses was broadly similar across groups (range 37.6–40.1 antibiotic courses per year of follow up). As expected, high prescription subjects accounted for a disproportionate amount of all antibiotic courses prescribed for their respective cohorts (T2DM 5.5%, T1DM 4.7%).

Among all individuals in the high prescription cohort, the six main ‘dominant’ antibiotics prescribed were macrolides, trimethoprim, penicillin, nitrofurantoin, tetracyclines, and first generation cephalosporins (Figure S6). The distribution of these main classes was broadly similar across diabetes groups (Figure S7).

### Duration of antibiotic courses in acute infections

During the study period, 110 751 people received at least one UTI code, 92 108 received a cellulitis code, and 232 030 a CAP code (Table S9). Analysis of antibiotic prescription duration did not show any clinically significant differences in median course length between groups (Table 5). When two antibiotics were prescribed for CAP on the same day, there were no clear differences in antibiotic combinations between groups (Table S10).

**Table 5.** Duration (days) of first antibiotic course prescribed within 7 days of an acute infection event

Urinary tract infections (UTI)		T1DM		T2DM	
Antibiotic	Sex	Case	Control	Case	Control
Amoxicillin	Both	7 (5–7)	7 (5–7)	7 (5–7)	7 (5–7)
Co-amoxiclav	Both	7 (7–7)	7 (7–7)	7 (7–7)	7 (7–7)
Fosfomycin	Both	NA	NA	1 (1–1)	1 (1–1)
Nitrofurantoin	Female	5 (3–7)	4 (3–7)	5 (3–7)	5 (3–7)
Nitrofurantoin	Male	7 (7–7)	7 (7–7)	7 (7–7)	7 (7–7)
Pivmecillinam	Both	5 (3–7)	7 (4–7)	5 (3–7)	5 (3–7)
Trimethoprim	Female	5 (3–7)	3 (3–7)	5 (3–7)	5 (3–7)
Trimethoprim	Male	7 (7–7)	7 (7–7)	7 (7–7)	7 (7–7)

Cellulitis		T1DM		T2DM	
Antibiotic	Sex	Case	Control	Case	Control
Clarithromycin	Both	7 (7–7)	7 (7–7)	7 (7–7)	7 (7–7)
Clindamycin	Both	7 (6.25–7)	7 (6–7)	7 (7–7)	7 (7–7)
Co-amoxiclav	Both	7 (7–7)	7 (7–7)	7 (7–7)	7 (7–7)
Doxycycline	Both	7 (7–14)	7 (7–7)	7 (7–13)	7 (7–13)
Erythromycin	Both	7 (5–7)	7 (7–10.5)	7 (7–7)	7 (7–7)
Flucloxacillin	Both	7 (7–7)	7 (7–7)	7 (7–7)	7 (7–7)

Community acquired pneumonia (CAP)		T1DM		T2DM	
Antibiotic	Sex	Case	Control	Case	Control
Amoxicillin	Both	7 (5–7)	7 (5–7)	7 (7–7)	7 (7–7)
Clarithromycin	Both	7 (7–7)	7 (7–7)	7 (7–7)	7 (7–7)
Doxycycline	Both	7 (7–7)	7 (7–7)	7 (7–7)	7 (7–7)
Erythromycin	Both	7 (7–7)	7 (5–7)	7 (7–7)	7 (7–7)

Results presented as median days, with interquartile range in brackets. T1DM, type 1 diabetes; T2DM, type 2 diabetes.

## Discussion

### Principal findings

In a large English primary care database, we have shown that people with diabetes have higher antibiotic prescription rates compared to the general population, even when accounting for recorded infection events and other confounders. This is true for both first-line, lower-risk ‘Access’ antibiotics as well as broader spectrum, higher-risk ‘Watch’ antibiotics.

### Strengths and weaknesses of the study

Our study has a number of strengths. We utilized a primary care database which allowed us to identify large T1DM and T2DM cohorts, providing substantial statistical power. The CPRD is broadly representative of the general population, including for age, sex, and IMD.^13^ Secondly, we reviewed all infection codes recorded in our cohorts and generated lists of bacterial and possibly bacterial infections, allowing for more methodologically rigorous infection counts than controlling for total infections alone. We used the English antibiotic AWaRe categories from the time of the study, rather than the WHO categories, to ensure our findings were relevant to the local healthcare context.^19^ The use of AWaRe categories in our pharmacoepidemiological study design is a relatively novel approach which is relevant to national antimicrobial policy objectives. Finally, we investigated multiple aspects of antibiotic prescribing, including course counts, course duration, and long-term prescriptions, to fully explore antibiotic exposure in this population.

Our study has a number of limitations. We did not have access to microbiological sensitivity results for individuals in our cohorts, or data about local AMR patterns. As these factors may influence prescribing choices, we could not determine the appropriateness of particular prescriptions. As with all observational research, the elevated prescription rates seen in our diabetes cohorts may still be due to residual confounding, despite the extensive adjustment in our analyses. Our analysis of antibiotic utilization was limited to the primary care setting only, but individuals may have received further unmeasured antibiotic courses from secondary care settings like hospitals. Our data relate to the UK, which is a high income setting and therefore our results may not generalize to low- and middle-income countries. Accurate infection coding is an established issue in UK primary care, where more than a third of antibiotic prescriptions are not linked to a medical condition.^25^ This makes it difficult to determine whether the increased prescription rates are due to a need for repeated courses of antibiotics for the same infection, or unmeasured infection burden beyond that which is recorded. Our study did not account for underlying disease severity or glycaemic control within the cohorts with diabetes, which is known to affect infection risk.^26^ We also acknowledge that only accounting for the core 12 QOF chronic comorbid conditions may contribute to unmeasured confounding.

### Findings in the context of what is known

Increased infection risk among individuals with diabetes is well-described,^5–8^ but few studies have examined antibiotic utilization in the same population.

A 2016 Danish cohort study of 150 000 individuals with T2DM found 62% received an antibiotic during 1.1 years of follow-up, versus 55% of matched controls.^27^ Our higher proportions (74% versus 62%) likely reflect a longer follow-up period (median 4.3 years). Their reported prescription rates (363 versus 275 per 1000 person years; adjusted RR 1.24) were lower than our own, although direct comparison is limited. Firstly, the Danish study used incident rather than prevalent cases of diabetes. Secondly, they analysed time to first antibiotic using a Cox model, which may underestimate true prescribing rates. A 2017 UK study using The Health Improvement Network (THIN) database reported an adjusted RR of 1.47 for antibiotic prescribing among people with diabetes versus those without.^28^ This study did not differentiate T1DM and T2DM, types of antibiotics, or report ethnicity in their analysis.

Stratified analyses showed higher prescription rates among females, consistent with higher infection rates previously observed in females in both diabetes groups.^5,7^ Higher antibiotic use in more deprived areas has been observed previously.^29,30^ We found higher prescription rates in White patients with diabetes compared to the non-White groups. While this partly reflects older age distributions in the White groups and the strong association between age and antibiotic use,^29,31^ age-ethnicity stratification showed persistently higher rates in White patients with T2DM. Potential explanations include practice-level prescribing variation, previously observed differences in antibiotic prescribing by ethnicity,^32^ age-related comorbidity profiles,^33^ or unmeasured differences in access to healthcare, health-seeking behaviours, and systemic biases related to diagnosis and prescribing.

The antibiotic classes most frequently seen in our ‘high-prescription’ cohort align with infection sites where elevated diabetes-related risk has been demonstrated.^6^ These included macrolides (azithromycin for recurrent chest infections), trimethoprim/nitrofurantoin (UTI prevention), penicillin (post-splenectomy prophylaxis), and tetracyclines (for acne, rosacea, COPD).

### Implications for research, policy, and practice

To our knowledge, this is the first study to examine antibiotic utilization in people with diabetes using the AWaRe classification. Although antibiotic use was higher in diabetes, Access antibiotics still represented most prescriptions (72% for T2DM, 66% for T1DM), exceeding the WHO's 60% benchmark^34^ and the European Union's 65% target.^35^ The UK's more ambitious 70% goal by 2030 was achieved by the T2DM cohort during the study period.^12^ Continued monitoring of antibiotic use in high-risk groups will be important in assessing the success of local and national stewardship policies, and pharmacoepidemiological studies can support this.

People with diabetes also received more Watch antibiotics compared to the general population. As these agents carry greater resistance potential, clinicians should prioritize Access antibiotics where appropriate and use microbiological sampling to support targeted therapy and monitor AMR development. Awareness of the broader adverse effects arising from cumulative antibiotic exposure, including infection with Clostridium difficile, organ dysfunction, and hypersensitivity reactions, remains similarly important.

Reducing antibiotic exposure requires optimization of diabetes care, given poor glycaemic control is associated with the risk of serious infections.^26^ Regular diabetes reviews from appropriate specialists, self-monitoring of glucose levels, and proactive treatment of infections remain central strategies.

### Conclusions and future perspectives

People with diabetes are exposed to higher levels of antibiotics, including those with the potential to drive antimicrobial resistance, which is a major global public health concern. Future work should investigate secondary care prescribing of antibiotic in people with diabetes, assess AMR in this population, integrate infection risk and antibiotic exposure into clinical guidelines, and evaluate stewardship initiatives aimed at clinicians caring for people with diabetes.

## Supplementary Material

dlag074_Supplementary_Data

## Data Availability

The data that support the findings of this study are available from Clinical Practice Research Datalink (CPRD) obtained under licence from the UK Medicines and Healthcare Products Regulatory Agency (MHRA), but restrictions apply to the availability of these data, which were used under licence for the current study and therefore are not publicly available. CPRD data governance and the licence to use CPRD data do not allow distribution of patient data directly to other parties. Researchers must apply directly to CPRD for data access (https://www.cprd.com).

## Supplementary data

Figures S1 to S7 and Tables S1 to S10 are available as Supplementary data at *JAC-AMR* Online.
