# Supplementary material for: Antibiotic prescribing in people with and without diabetes: a population-based matched cohort study
Source: JAC Antimicrob Resist. 2026 May 8;8(3):dlag074. doi: 10.1093/jacamr/dlag074 (PMC13155108; doi:10.1093/jacamr/dlag074)
Supplement: dlag074_Supplementary_Data [file dlag074_supplementary_data.docx]

**Title:** Antibiotic exposure and prescription rates in type 1 and type 2 diabetes: a population-based matched cohort study

**Supplementary material**

Table of Contents

[Diabetes definition, cohort selection, and variable definitions 3](#_Toc208995959)

[**Supplementary Figure S1.** Flowchart of selection of patients with type 2 diabetes, type 1 diabetes, and matched controls 3](#_Toc208995960)

[**Supplementary Figure S2.** Algorithm to determine diabetes type 4](#_Toc208995961)

[**Supplementary Table S1.** Diabetes, infection, antibiotic and ethnicity variable code definitions 5](#_Toc208995962)

[Classification of antibiotics and infection Read codes 6](#_Toc208995963)

[**Supplementary Table S2.** Classification of antibiotic products by antibiotic class and AWaRe England status (2019 version) prescribed during study period 6](#_Toc208995964)

[**Supplementary Table S3.** Read codes for urinary tract infection (UTI), cellulitis, and community-acquired pneumonia 9](#_Toc208995965)

[**Supplementary Table S4.** National Institute for Health and Care Excellence (NICE)-recommended antibiotics to treat cellulitis, urinary tract infection (UTI), and community-acquired pneumonia (CAP) 12](#_Toc208995966)

[Antibiotic course counts, rates, and rate ratios 14](#_Toc208995967)

[**Supplementary Figure S3.** Age distribution by ethnicity in the (A) T1DM cohort and (B) T2DM cohort 14](#_Toc208995968)

[**Supplementary Table S5**. Course counts, rates, and rate ratios for young (age <50 years) T2DM subjects 15](#_Toc208995969)

[**Supplementary Table S6.** Antibiotic course counts, rates (per 1000 person-years), and unadjusted rate ratios, for people with T1DM and T2DM compared with age-sex-practice-matched controls, stratified by antibiotic class 16](#_Toc208995970)

[**Supplementary Table S7.** Breakdown of Reserve antibiotics prescribed to each group. Results presented as number of courses, with proportion of all Reserve courses for that diabetes group in brackets 18](#_Toc208995971)

[**Supplementary Figure S4.** Antibiotic prescription rates for people with T1DM and matched controls, stratified by age group and ethnicity 19](#_Toc208995972)

[**Supplementary Figure S5.** Antibiotic prescription rates for people with T2DM and matched controls, stratified by age group and ethnicity 20](#_Toc208995973)

[High prescription count analyses 21](#_Toc208995974)

[**Supplementary Table S8.** Number and proportion of ‘high prescription’ individuals* in each diabetes group 21](#_Toc208995975)

[**Supplementary Figure S6.** Dominant antibiotic class among all ‘high prescription’ individuals 22](#_Toc208995976)

[**Supplementary Figure S7.** Dominant antibiotic class among high-prescription individuals, stratified by diabetes group 23](#_Toc208995977)

[Acute infection antibiotic prescription analyses 24](#_Toc208995978)

[**Supplementary Table S9.** Number of subjects with at least one UTI, cellulitis, or community acquired pneumonia (CAP) infection code during study follow up, and total UTI infection code episodes 24](#_Toc208995979)

[**Supplementary Table S10**. Combinations of community acquired pneumonia antibiotics prescribed on the same day following acute lower respiratory tract infection event. Results presented as number of instances of each combination per group, with percentage denoting the proportion each combination represents within each patient cohort. 25](#_Toc208995980)

## **Diabetes definition, cohort selection, and variable definitions**

### **Supplementary Figure S1.** Flowchart of selection of patients with type 2 diabetes, type 1 diabetes, and matched controls

*****Reasons for exclusion: type unclassifiable or presumed dead

### **Supplementary Figure S2.** Algorithm to determine diabetes type

### **Supplementary Table S1.** Diabetes, infection, antibiotic and ethnicity variable code definitions

| **Variable to be classified** | **Coding definition** | **Further requirement** |
| --- | --- | --- |
| Type 2 Diabetes | Read code in primary care record for diabetes recorded prior to 01/01/2015 | Classification of type 2 determined by Read codes, history of anti-diabetic medication, and age at diagnosis – See Supplementary Figure 2 |
| Type 1 Diabetes | Read code in primary care record for diabetes recorded prior to 01/01/2015 | Classification of type 1 determined by Read codes, history of anti-diabetic medication, and age at diagnosis – See Supplementary Figure 2 |
| Ethnicity | Read code in primary care anywhere on the patient’s record | Classified as “White”, “South Asian”, “Black” if only codes from these groups appear.  Classified as “Mixed/Other” if “Mixed/Other” or other permutation of codes present |
| Infection | Read code for infection recorded on primary care record during follow-up period (01/01/2015 to 31/12/2019) | No repeat of the same infection code allowed within the next 90 days  Individual infection codes classified as ‘bacterial’, ‘viral’, ‘fungal’, ‘mycobacterial’ or ‘other’, or ‘possibly bacterial’ if a non-specific infection/presentation code could reasonably be caused by a bacterium but also by another infectious agent |
| Antibiotic | Read code for antibiotic prescription during follow-up period (01/01/2015 – 31/12/2019) | Antibiotics were further classified by 1) their pharmaceutical class and 2) their AWaRe status (2019 England adapted version)^1^ |

^1^ Emma Budd, Emma Cramp, Mike Sharland, Kieran Hand, Philip Howard, Peter Wilson, Mark Wilcox, Berit Muller-Pebody, Susan Hopkins, Adaptation of the WHO Essential Medicines List for national antibiotic stewardship policy in England: being AWaRe, *Journal of Antimicrobial Chemotherapy*, Volume 74, Issue 11, November 2019, Pages 3384–3389, <https://doi.org/10.1093/jac/dkz321>

## **Classification of antibiotics and infection Read codes**

### **Supplementary Table S2.** Classification of antibiotic products by antibiotic class and AWaRe England status (2019 version) prescribed during study period

| **Drug substance name** | **Antibiotic class** | **AWaRe England Class 2019*** |
| --- | --- | --- |
| Amikacin sulfate | Aminoglycosides | Watch |
| Gentamicin sulfate | Aminoglycosides | Access |
| Neomycin sulfate | Aminoglycosides | Watch |
| Tobramycin | Aminoglycosides | Watch |
| Chloramphenicol | Amphenicols | Watch |
| Amoxicillin sodium/ Potassium clavulanate | Beta_lactam_beta_lactamase_inhibitors | Watch |
| Amoxicillin trihydrate/ Potassium clavulanate | Beta_lactam_beta_lactamase_inhibitors | Watch |
| Piperacillin sodium/ Tazobactam sodium | Beta_lactam_beta_lactamase_inhibitors_anti_pseudomonal | Watch |
| Cilastatin sodium/ Imipenem monohydrate | Carbapenems | Reserve |
| Ertapenem sodium | Carbapenems | Reserve |
| Meropenem trihydrate | Carbapenems | Reserve |
| Temocillin sodium | Carboxypenicillins | Watch |
| Cefadroxil monohydrate | First_generation_cephalosporins | Watch |
| Cefalexin | First_generation_cephalosporins | Watch |
| Cefradine | First_generation_cephalosporins | Watch |
| Cefradine | First_generation_cephalosporins | Watch |
| Ciprofloxacin | Fluoroquinolones | Watch |
| Ciprofloxacin hydrochloride | Fluoroquinolones | Watch |
| Ciprofloxacin lactate | Fluoroquinolones | Watch |
| Levofloxacin hemihydrate | Fluoroquinolones | Watch |
| Moxifloxacin hydrochloride | Fluoroquinolones | Watch |
| Nalidixic acid | Fluoroquinolones | Not included |
| Norfloxacin | Fluoroquinolones | Watch |
| Ofloxacin | Fluoroquinolones | Watch |
| Dalbavancin hydrochloride | Glycopeptides | Reserve |
| Teicoplanin | Glycopeptides | Watch |
| Vancomycin hydrochloride | Glycopeptides | Watch |
| Tigecycline | Glycylcyclines | Reserve |
| Metronidazole | Imidazoles | Access |
| Metronidazole benzoate | Imidazoles | Access |
| Tinidazole | Imidazoles | Not included |
| Clindamycin hydrochloride | Lincosamides | Watch |
| Clindamycin phosphate | Lincosamides | Watch |
| Daptomycin | Lipopeptides | Reserve |
| Fidaxomicin | Macrocyclics | Watch |
| Azithromycin | Macrolides | Watch |
| Azithromycin dihydrate | Macrolides | Watch |
| Clarithromycin | Macrolides | Watch |
| Erythromycin | Macrolides | Watch |
| Erythromycin ethyl succinate | Macrolides | Watch |
| Erythromycin stearate | Macrolides | Watch |
| Aztreonam lysine | Monobactams | Reserve |
| Nitrofurantoin | Nitrofurantoin | Access |
| Linezolid | Oxazolidinones | Reserve |
| Amoxicillin sodium | Penicillins | Access |
| Amoxicillin trihydrate | Penicillins | Access |
| Ampicillin | Penicillins | Access |
| Ampicillin sodium | Penicillins | Access |
| Ampicillin sodium/ Flucloxacillin sodium | Penicillins | Access |
| Ampicillin trihydrate/ Flucloxacillin magnesium | Penicillins | Access |
| Ampicillin trihydrate/ Flucloxacillin sodium | Penicillins | Access |
| Benzathine benzylpenicillin | Penicillins | Access |
| Benzylpenicillin sodium | Penicillins | Access |
| Flucloxacillin magnesium | Penicillins | Access |
| Flucloxacillin sodium | Penicillins | Access |
| Phenoxymethylpenicillin potassium | Penicillins | Access |
| Pivmecillinam hydrochloride | Penicillins | Access |
| Fosfomycin calcium | Phosphonics | Access |
| Fosfomycin sodium | Phosphonics | Reserve |
| Fosfomycin trometamol | Phosphonics | Access |
| Colistimethate sodium | Polymyxins | Reserve |
| Colistin sulfate | Polymyxins | Reserve |
| Rifampicin | Rifamycins | Watch |
| Rifaximin | Rifamycins | Watch |
| Cefaclor monohydrate | Second_generation_cephalosporins | Watch |
| Cefuroxime axetil | Second_generation_cephalosporins | Watch |
| Cefuroxime sodium | Second_generation_cephalosporins | Watch |
| Fusidic acid | Steroid_antibacterials | Access |
| Sodium fusidate | Steroid_antibacterials | Watch |
| Pristinamycin | Streptogramins | Watch |
| Sulfadiazine | Sulfonamides | Not included |
| Dapsone | Sulfone | Not included |
| Demeclocycline hydrochloride | Tetracyclines | Watch |
| Doxycycline hyclate | Tetracyclines | Access |
| Doxycycline monohydrate | Tetracyclines | Access |
| Lymecycline | Tetracyclines | Watch |
| Minocycline hydrochloride | Tetracyclines | Watch |
| Oxytetracycline dihydrate | Tetracyclines | Watch |
| Tetracycline hydrochloride | Tetracyclines | Access |
| Cefixime | Third_generation_cephalosporins | Watch |
| Cefotaxime sodium | Third_generation_cephalosporins | Watch |
| Ceftazidime pentahydrate | Third_generation_cephalosporins | Watch |
| Ceftriaxone sodium | Third_generation_cephalosporins | Watch |
| Trimethoprim | Trimethoprim | Access |
| Sulfamethoxazole/ Trimethoprim | Trimethoprim_sulfonamide_combinations | Access |

* Access: antibiotics with a narrow spectrum of activity, fewer side effects, lower costs, and lower antimicrobial resistance potential. They are first or second choice antibiotics recommended for empiric treatment of the most common infections. Watch: antibiotics which have a higher resistance potential and broader spectrum of action. They are first or second choice antibiotics for a limited number of infective syndromes, and their use should be carefully monitored. Reserve: ‘last resort’ antibiotics used for highly-selected patients (life threatening infections due to multi-drug-resistant bacteria) and are closely monitored.

### **Supplementary Table S3.** Read codes for urinary tract infection (UTI), cellulitis, and community-acquired pneumonia

| **Cellulitis codes** | | | |
| --- | --- | --- | --- |
| **Medcodeid** | **Term** | **Medcodeid** | **Term** |
| 117333013 | Cellulitis of toe | 399893016 | Cellulitis and abscess of digit NOS |
| 117539013 | Pyoderma | 399894010 | Other cellulitis and abscess |
| 117541014 | Pyogenic infection of skin and subcutis | 399895011 | Cellulitis and abscess of cheek (external) |
| 1232396011 | Cervical abscess | 399896012 | Cellulitis and abscess of cheek |
| 158680013 | Cellulitis of external ear | 399897015 | Cellulitis and abscess of nose (external) |
| 206541013 | Cellulitis of foot | 399898013 | Cellulitis and abscess of neck |
| 219471000000116 | Cellulitis, external ear | 399899017 | Cellulitis and abscess of hand excluding digits |
| 25168017 | Cellulitis of penis | 399900010 | Cellulitis and abscess of hand unspecified |
| 302270018 | Cellulitis of floor of mouth | 399901014 | Cellulitis and abscess of leg |
| 302314015 | Other oral epithelium disturbances | 399903012 | Cellulitis and abscess of foot excluding toe |
| 302316018 | Oral epithelium disturbances NOS | 399904018 | Cellulitis and abscess of head unspecified |
| 302317010 | Other and unspecified diseases of oral soft tissue | 399909011 | Cellulitis of digit |
| 303740015 | Disorder of oral soft tissues | 40768015 | Cellulitis of ankle |
| 308266016 | Cellulitis and abscess of finger and toe | 423153016 | Cellulitis of upper limb |
| 308269011 | Cellulitis and abscess of finger | 42341000006115 | Oral cellulitis and abscess |
| 308270012 | Cellulitis and abscess of finger unspecified | 450686015 | Cellulitis - anus or rectum |
| 308281010 | Cellulitis and abscess of finger NOS | 450687012 | Perianal cellulitis |
| 308282015 | Cellulitis and abscess of toe | 472876012 | Cutaneous cellulitis |
| 308283013 | Cellulitis and abscess of toe unspecified | 48067018 | Cellulitis of larynx |
| 308300010 | Cellulitis and abscess of face | 540621000006119 | Cellulitis and abscess NOS |
| 308307013 | Cellulitis and abscess of chin | 540851000006112 | Cellulitis and abscess of foot |
| 308308015 | Cellulitis and abscess of submandibular region | 540921000006112 | Cellulitis and abscess of hand |
| 308310018 | Cellulitis and abscess of temple region | 541011000006113 | Cellulitis and abscess of lower limb |
| 308311019 | Cellulitis of face | 541291000006111 | Cellulitis of face |
| 308312014 | Cellulitis and abscess of face NOS | 541421000006113 | Cellulitis of trunk |
| 308316012 | Cellulitis and abscess of trunk | 308344013 | Cellulitis and abscess of buttock |
| 308317015 | Cellulitis and abscess of chest wall | 308349015 | Cellulitis and abscess of hip |
| 308318013 | Cellulitis and abscess of breast | 308350015 | Cellulitis and abscess of thigh |
| 308319017 | Cellulitis and abscess of back | 308351016 | Cellulitis and abscess of knee |
| 308320011 | Cellulitis and abscess of abdominal wall | 308352011 | Cellulitis and abscess of lower leg |
| 308321010 | Cellulitis and abscess of umbilicus | 308353018 | Cellulitis and abscess of ankle |
| 308322015 | Cellulitis and abscess of flank | 308354012 | Cellulitis and abscess of leg NOS |
| 308323013 | Cellulitis and abscess of groin | 308358010 | Cellulitis and abscess of foot unspecified |
| 308324019 | Cellulitis and abscess of perineum | 308359019 | Cellulitis and abscess of heel |
| 308325018 | Cellulitis and abscess of trunk NOS | 308360012 | Cellulitis in diabetic foot |
| 308326017 | Cellulitis and abscess of upper limb | 308361011 | Cellulitis and abscess of foot NOS |
| 308327014 | Cellulitis and abscess of shoulder | 308363014 | Other specified cellulitis and abscess |
| 308328016 | Cellulitis and abscess of axilla | 308367010 | Cellulitis and abscess NOS |
| 308329012 | Cellulitis and abscess of upper arm | 308368017 | Cellulitis |
| 308330019 | Cellulitis and abscess of elbow | 308402015 | Pyoderma NOS |
| 308331015 | Cellulitis and abscess of forearm | 308446011 | Cellulitis of breast |
| 308342012 | Cellulitis and abscess of wrist | 308453019 | Cellulitis of skin |
| 308343019 | Cellulitis of hand | 32771017 | Cellulitis of axilla |
| 396325011 | Oral cellulitis and abscess NOS | 357316015 | Cellulitis of skin area excluding digits of hand or foot |
| 396332019 | Oral soft tissue diseases NOS | 357317012 | Cellulitis of dorsum of hand |
| 399892014 | Cellulitis and abscess of toe NOS | 357321017 | Cellulitis of palm of hand |
| 58130016 | Cellulitis of scrotum | 357328011 | Cellulitis of lower limb |
| 61479010 | Onychia of finger | 359601000006118 | Abscess of neck |
| 62096018 | Cellulitis of neck | 370601000006113 | [X]Cellulitis of other parts of limb |
| 78133018 | Cellulitis of trunk | 370621000006115 | [X]Cellulitis of thumb |
| 85030014 | Cellulitis of lip | 889041000006116 | Cellulitis/abscess - buttock |
| 888961000006114 | Cellulitis - toe | 889051000006119 | Cellulitis - leg - excl. foot |
| 888981000006116 | Cellulitis/abscess NOS | 889061000006117 | Cellulitis - foot - excl. toe |
| **Urinary tract infection (UTI) codes** | | | |
| 113884018 | Urinary tract infection | 305203014 | [X]Other cystitis |
| 304206012 | Other specified cystitis | 3615701000006117 | UTI - Urinary tract infection |
| 304208013 | Other cystitis NOS | 65119018 | Cystitis |
| 304209017 | Cystitis NOS | 74781000006117 | Urinary tract infection, site not specified |
| 304323018 | Urinary tract infection, site not specified NOS | 886811000006115 | Urinary tract infection NOS |
| **Community acquired pneumonia codes** | | | |
| 301811017 | [X]Other bacterial pneumonia | 219771000006116 | Legionella pneumonia |
| 301818011 | [X]Other pneumonia, organism unspecified | 1772681000006119 | Lobar pneumonia |
| 301812012 | [X]Pneumonia due to other specified infectious organisms | 301409015 | Lobar pneumonia |
| 457801000006117 | Acute lower respiratory tract infection | 5887681000006112 | Lower lobe pneumonia |
| 546411000006111 | Acute lower respiratory tract infection | 3316381000006114 | Lower respiratory infection |
| 579878017 | Acute lower respiratory tract infection | 396090018 | Lower respiratory tract infection |
| 350051017 | Atypical pneumonia | 3316401000006114 | LRTI - Lower respiratory tract infection |
| 301376012 | Bacterial pneumonia | 78272011 | Mycoplasma pneumonia |
| 1772711000006118 | Basal pneumonia | 2164029012 | Mycoplasmal pneumonia |
| 1772771000006110 | Bronchopneumonia | 301368010 | Other bacterial pneumonia |
| 1222332017 | Chest infection - other bacterial pneumonia | 739941000006111 | Pneumococcal lobar pneumonia |
| 546451000006112 | Chest infection - pneumococcal pneumonia | 301817018 | Pneumonia |
| 1222333010 | Chest infection - pneumonia organism OS | 301375011 | Pneumonia due to bacteria NOS |
| 350054013 | Chlamydial pneumonia | 2765492018 | Pneumonia due to Gram negative bacteria |
| 1479355018 | Community acquired pneumonia | 219851000006115 | Pneumonia due to haemophilus influenzae |
| 1787121000006116 | Community acquired pneumonia | 107173015 | Pneumonia due to Klebsiella pneumoniae |
| 633531000006116 | E.coli pneumonia | 301370018 | Pneumonia due to other specified bacteria |
| 2671161016 | Escherichia coli pneumonia | 301377015 | Pneumonia due to other specified organisms |
| 219841000006117 | Haemophilus influenzae pneumonia | 301382010 | Pneumonia due to specified organism NOS |
| 7044731000006115 | HAP - hospital acquired pneumonia | 219991000006118 | Pneumonia due to streptococcus, group B |
| 1787131000006118 | Hospital acquired pneumonia | 885241000006112 | Pneumonia NOS |
| 2674072012 | Hospital acquired pneumonia | 396104016 | Pneumonia with infectious diseases EC |
| 301408011 | Infective pneumonia | 301404013 | Pneumonia with infectious diseases EC NOS |
| 1229740013 | Influenza with bronchopneumonia | 301403019 | Pneumonia with other infectious diseases EC NOS |
| 13423018 | Legionella infection | 459416013 | Postoperative pneumonia |
| 219971000006119 | Staphylococcal pneumonia | 69026013 | Pseudomonal pneumonia |
| 56816017 | Streptococcal pneumonia |  |  |

### **Supplementary Table S4.** National Institute for Health and Care Excellence (NICE)-recommended antibiotics to treat cellulitis, urinary tract infection (UTI), and community-acquired pneumonia (CAP)

| **Cellulitis** | | |
| --- | --- | --- |
|  | **Antibiotic choice** | **NICE-recommended duration** |
| First line | Flucloxacillin 500-1000mg QDS | 5-7 days then review |
| First line in penicillin allergy | Clarithromycin 500mg BD | 5-7 days then review |
|  | Erythromycin (in pregnancy) 500mg QDS | 5-7 days then review |
|  | Doxycycline 200mg on the first day then 100mg OD | 5-7 days then review |
| First line if near eyes or nose^ | Co-amoxiclav 500/125mg TDS | 5-7 days then review |
| First line if near eyes or nose + penicillin allergy^ | Clarithromycin 500mg BD with metronidazole 400mg TDS | 5-7 days then review |
| Second line | Co-amoxiclav 500/125mg TDS | 5-7 days then review |
|  | Clindamycin 150-300mg QDS | 7 days then review |
| **UTI - women** | | |
|  | **Antibiotic choice** | **NICE-recommended duration** |
| First line | Nitrofurantoin 100mg BD | 3 days |
|  | Trimethoprim 200mg BD | 3 days |
| Second line | Nitrofurantoin (if not used first line) 100mg BD | 3 days |
|  | Pivmecillinam (400mg initial then 200mg TDS) | 3 days |
|  | Fosfomycin 3g single sachet | Single dose |
| **UTI - men** | | |
|  | **Antibiotic choice** | **NICE-recommended duration** |
| First line | Nitrofurantoin 100mg BD | 7 days |
|  | Trimethoprim 200mg BD | 7 days |
| Second line | Nitrofurantoin (if not used first line) 100mg BD | 3 days |
| **Community-acquired pneumonia** | | |
|  | **Antibiotic choice** | **NICE-recommended duration** |
| First line (CRB-65=0)* | Amoxicillin 500mg TDS | 5 days |
| First line (penicillin allergy) (CRB-65=0)* | Doxycycline 200mg on first day then 100mg OD | 5 days |
|  | Clarithromycin 500mg twice daily | 5 days |
|  | Erythromycin 500mg four times a day (in pregnancy) | 5 days |
| First line (CRB-65=1-2)* | Amoxicillin 500mg TDS + Clarithromycin 500mg BD *or* Erythromycin 500mg QDS | 5 days |
| First line (penicillin allergy) (CRB-65=1-2)* | Doxycycline 200mg on first day then 100mg OD *or*  Clarithromycin 500mg BD | 5 days |

OD = once daily, BD = twice daily, TDS = three times daily, QDS = four times daily

^Infection codes for cellulitis of eyes or nose excluded for this analysis

*****The CRB-65 score is a clinical prediction tool which is validated for predicting mortality from community acquired pneumonia, based on four clinical parameters: confusion, respiratory rate, blood pressure, and age (1). An additional parameter, urea level, is incorporated into the full CURB-65 score, but is not commonly used in primary care due to lack of blood test availability. It is recommended by NICE for risk-stratifying patients presenting with CAP in primary care, and antibiotic recommendation varies depending on CURB result. A score of 0 indicates a low mortality risk, and likely suitable for community treatment. A score of 1-2 indicates a moderate risk of mortality, but the patient may still be appropriate for community treatment. Higher scores require referral to hospital.

## **Antibiotic course counts, rates, and rate ratios**

### **Supplementary Figure S3.** Age distribution by ethnicity in the (A) T1DM cohort and (B) T2DM cohort


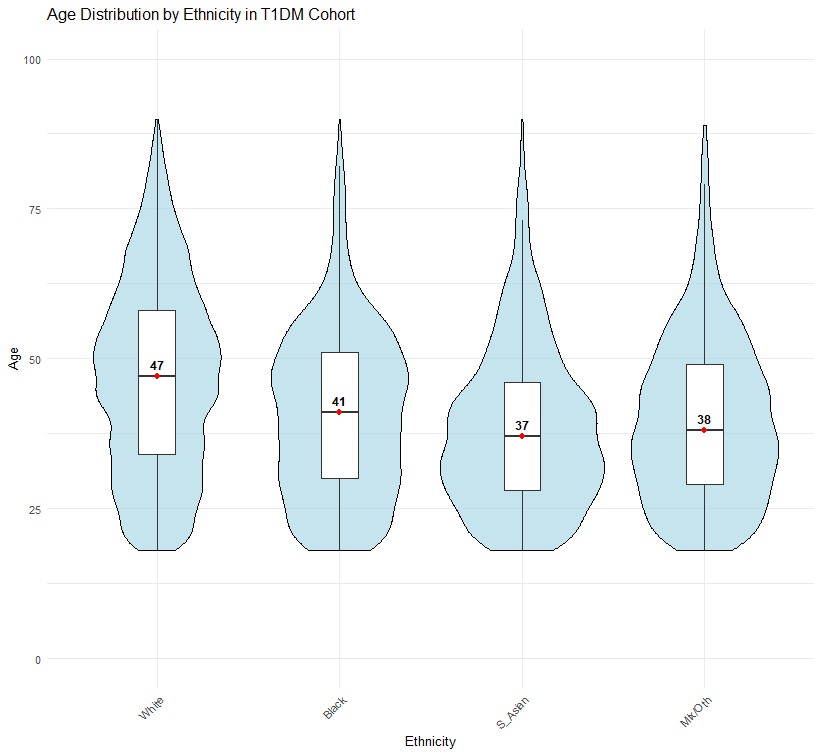

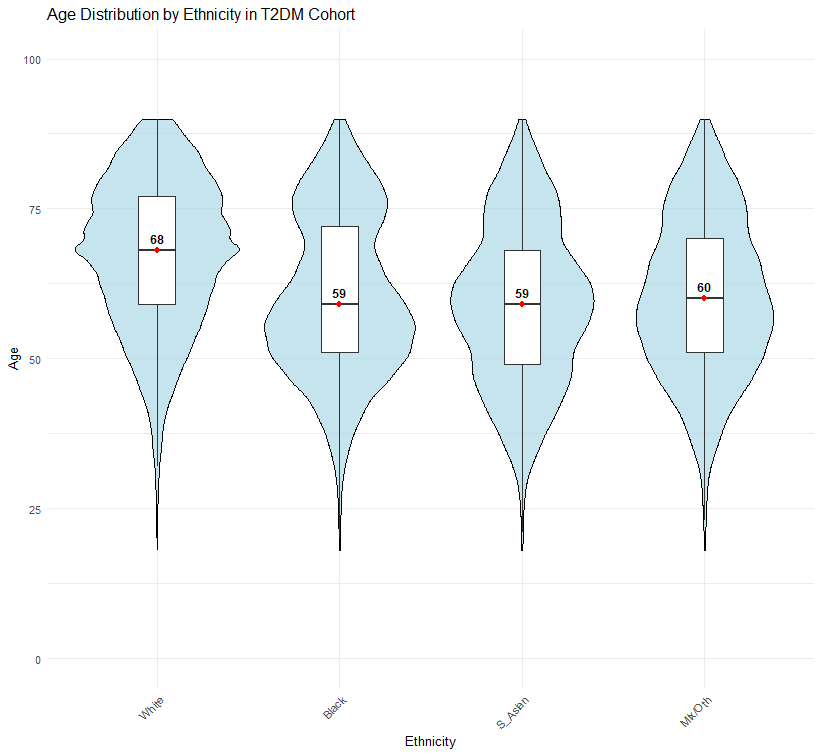


A

B

### **Supplementary Table S5**. Course counts, rates, and rate ratios for young (age <50 years) T2DM subjects

|  | **Controls**  **(n = 118,209)** | | **T2DM**  **(n = 59,159)** | | **Rate ratios** | | |
| --- | --- | --- | --- | --- | --- | --- | --- |
|  | **Courses** | **Rate†** | **Courses** | **Rate†** | **Unadjusted*** | **Adjusted ^(1)^** | **Adjusted ^(2)^** |
| **All antibiotics** | 219,039 | 425.71 | 246,790 | 956.80 | 2.25 (2.24-2.27) | 1.64 (1.63-1.66) | 1.39 (1.38-1.40) |
| **Access** | 158,972 | 308.97 | 173,008 | 670.75 | 2.17 (2.16-2.19) | 1.61 (1.59-1.62) | 1.35 (1.33-1.36) |
| **Watch** | 62,184 | 120.86 | 76,142 | 295.20 | 2.46 (2.43-2.48) | 1.73 (1.70-1.75) | 1.49 (1.46-1.51) |
| **†** Per 1000 person-years  ***** Conditioned on match sets (age, sex, and GP practice matched)  **(1)** Adjusted for BMI, ethnicity, IMD, smoking status, and comorbidity count  **(2)** Adjusted for BMI, ethnicity, IMD, smoking status, comorbidity count, and infection count (bacterial + possibly bacterial infections) | | | | | | | |

### **Supplementary Table S6.** Antibiotic course counts, rates (per 1000 person-years), and unadjusted rate ratios, for people with T1DM and T2DM compared with age-sex-practice-matched controls, stratified by antibiotic class

|  | T1DM | | | T2DM | | |
| --- | --- | --- | --- | --- | --- | --- |
|  | **Cases** | **Controls** | **Rate ratio (95% CI)** | **Cases** | **Controls** | **Rate ratio (95% CI)** |
| Antibiotic | **Courses (Rate)** | **Courses (Rate)** |  | **Courses (Rate)** | **Courses (Rate)** |  |
| All antibiotics | 160081 (1120.52) | 151238 (518.61) | 2.21 (2.19-2.22) | 2918090 ( 1316.47 ) | 3805948 ( 853.45 ) | 1.57 (1.57-1.57) |
| Penicillins | 57375 ( 401.61 ) | 57562 ( 197.39 ) | 2.07 (2.05-2.09) | 1044558 ( 471.24 ) | 1354539 ( 303.74 ) | 1.57 (1.57-1.58) |
| Tetracyclines | 19996 ( 139.97 ) | 22699 ( 77.84 ) | 1.83 (1.79-1.87) | 383122 ( 172.84 ) | 526278 ( 118.01 ) | 1.49 (1.48-1.49) |
| Macrolides | 19325 ( 135.27 ) | 15993 ( 54.84 ) | 2.50 (2.45-2.56) | 311778 ( 140.66 ) | 427732 ( 95.91 ) | 1.50 (1.50-1.51) |
| Nitrofurantoin | 13497 ( 94.48 ) | 14261 ( 48.9 ) | 1.98 (1.93-2.03) | 304499 ( 137.37 ) | 408791 ( 91.67 ) | 1.52 (1.51-1.53) |
| Trimethoprim | 10181 ( 71.26 ) | 11129 ( 38.16 ) | 1.97 (1.92-2.03) | 257381 ( 116.12 ) | 320746 ( 71.92 ) | 1.67 (1.66-1.68) |
| Beta_lactam_beta_lactamase_inhibitors | 8070 ( 56.49 ) | 5830 ( 19.99 ) | 2.93 (2.83-3.04) | 128540 ( 57.99 ) | 154651 ( 34.68 ) | 1.72 (1.71-1.74) |
| Fluoroquinolones | 5403 ( 37.82 ) | 3825 ( 13.12 ) | 2.95 (2.83-3.08) | 78740 ( 35.52 ) | 103436 ( 23.19 ) | 1.58 (1.57-1.60) |
| First_generation_cephalosporins | 5401 ( 37.81 ) | 2758 ( 9.46 ) | 4.11 (3.92-4.31) | 107350 ( 48.43 ) | 113435 ( 25.44 ) | 1.99 (1.97-2.01) |
| Steroid_antibacterials | 4563 ( 31.94 ) | 4176 ( 14.32 ) | 2.24 (2.14-2.34) | 76302 ( 34.42 ) | 94329 ( 21.15 ) | 1.64 (1.63-1.66) |
| Amphenicols | 4253 ( 29.77 ) | 4619 ( 15.84 ) | 1.93 (1.85-2.02) | 92376 ( 41.67 ) | 133311 ( 29.89 ) | 1.41 (1.39-1.42) |
| Imidazoles | 3839 ( 26.87 ) | 5139 ( 17.62 ) | 1.53 (1.46-1.59) | 63120 ( 28.48 ) | 96167 ( 21.56 ) | 1.35 (1.34-1.36) |
| Trimethoprim_sulfonamide_combinations | 3332 ( 23.32 ) | 878 ( 3.01 ) | 7.54 (6.99-8.13) | 19587 ( 8.84 ) | 22568 ( 5.06 ) | 1.85 (1.81-1.89) |
| Lincosamides | 1977 ( 13.84 ) | 1084 ( 3.72 ) | 3.70 (3.43-3.99) | 20903 ( 9.43 ) | 17672 ( 3.96 ) | 2.46 (2.41-2.51) |
| Sulfone | 815 ( 5.7 ) | 345 ( 1.18 ) | 6.20 (5.43-7.08) | 2970 ( 1.34 ) | 4390 ( 0.98 ) | 1.33 (1.26-1.39) |
| Polymyxins | 680 ( 4.76 ) | 74 ( 0.25 ) | 21.32 (16.61-27.37) | 3179 ( 1.43 ) | 6092 ( 1.37 ) | 1.02 (0.98-1.07) |
| Rifamycins | 480 ( 3.36 ) | 391 ( 1.34 ) | 2.34 (2.05-2.68) | 12541 ( 5.66 ) | 8734 ( 1.96 ) | 3.07 (2.98-3.16) |
| Aminoglycosides | 355 ( 2.48 ) | 250 ( 0.86 ) | 2.90 (2.45-3.42) | 3723 ( 1.68 ) | 5235 ( 1.17 ) | 1.41 (1.35-1.47) |
| Second_generation_cephalosporins | 176 ( 1.23 ) | 68 ( 0.23 ) | 5.75 (4.31-7.66) | 2356 ( 1.06 ) | 2289 ( 0.51 ) | 2.12 (2.00-2.25) |
| Phosphonics | 125 ( 0.87 ) | 92 ( 0.32 ) | 2.87 (2.17-3.79) | 3072 ( 1.39 ) | 3381 ( 0.76 ) | 1.88 (1.79-1.98) |
| Glycopeptides | 80 ( 0.56 ) | 24 ( 0.08 ) | 8.69 (5.33-14.15) | 947 ( 0.43 ) | 1078 ( 0.24 ) | 1.92 (1.75-2.10) |
| Carbapenems | 79 ( 0.55 ) | <5 | 35.13 (12.69-97.25) | 99 ( 0.04 ) | 74 ( 0.02 ) | 2.88 (2.10-3.93) |
| Third_generation_cephalosporins | 38 ( 0.27 ) | 33 ( 0.11 ) | 2.06 (1.28-3.31) | 663 ( 0.3 ) | 720 ( 0.16 ) | 2.08 (1.86-2.32) |
| Oxazolidinones | 19 ( 0.13 ) | <5 | 12.82 (3.74-43.98) | 189 ( 0.09 ) | 139 ( 0.03 ) | 3.29 (2.61-4.15) |
| Monobactams | 14 ( 0.1 ) | 0 | NA | <5 | 0 | NA |
| Beta_lactam_beta_lactamase_inhibitors_anti_pseudomonal | <5 | 0 | NA | 24 ( 0.01 ) | 30 ( 0.01 ) | 1.60 (0.91-2.82) |
| Streptogramins | <5 | 0 | NA | 0 | <5 | NA |
| Sulfonamides | <5 | <5 | 1.58 (0.10-25.79) | 24 ( 0.01 ) | 81 ( 0.02 ) | 0.92 (0.57-1.49) |
| Glycylcyclines | <5 | 0 | NA | 6 (0) | <5 | 5.29 (1.26-22.31) |
| Macrocyclics | <5 | 0 | NA | 17 ( 0.01 ) | 36 ( 0.01 ) | 0.90 (0.49-1.65) |
| Carboxypenicillins | 0 | 0 | NA | <5 | <5 | 1.52 (0.09-24.98) |
| Lipopeptides | 0 | 0 | NA | 20 ( 0.01 ) | 7 (0) | 7.23 (2.88-18.17) |
| Access | 106146 (742.99) | 109525 (375.58) | 2.02 (2.00-2.04) | 2098960 ( 946.93 ) | 2764199 ( 619.85 ) | 1.55 (1.55-1.56) |
| Watch | 54162 (379.12) | 42948 (147.27) | 2.62 (2.59-2.65) | 854098 ( 385.32 ) | 1082435 ( 242.73 ) | 1.63 (1.62-1.63) |
| Reserve | 794 (5.56) | 81 (0.28) | 22.17 (17.49-28.11) | 3529 ( 1.59 ) | 6356 ( 1.43 ) | 1.10 (1.05-1.15) |

### **Supplementary Table S7.** Breakdown of Reserve antibiotics prescribed to each group. Results presented as number of courses, with proportion of all Reserve courses for that diabetes group in brackets

|  | **T2DM** | | **T1DM** | |
| --- | --- | --- | --- | --- |
|  | **Cases** | **Controls** | **Cases** | **Controls** |
| **Colistin** | 3205 (90.8%) | 6126 (96.4%) | 681 (85.8%) | 74 (91.4%) |
| Inhaled colistin | 3205 (90.8%) | 6125 (96.4%) | 681 (85.8%) | 74 (91.4%) |
| Tablet colistin | - | <5 | - | - |
| **Meropenem** | 33 (0.9%) | 13 (0.2%) | 78 (9.8%) | <5 |
| **Linezolid** | 193 (5.5%) | 141 (2.2%) | 19 (2.4%) | <5 |
| **Daptomycin** | 20 (0.6%) | 8 (0.1%) | - | - |
| **Aztreonam** | <5 | - | 14 (1.8%) | - |
| **Imipenem** | - | <5 | - | - |
| **Dalbavancin** | <5 | - | - | - |
| **Ertapenem** | 67 (1.9%) | 63 (1%) | <5 | - |
| **Injected Fosfomycin** | - | <5 | - | - |
| **Tigecycline** | <5 | <5 | <5 | - |

### **Supplementary Figure S4.** Antibiotic prescription rates for people with T1DM and matched controls, stratified by age group and ethnicity


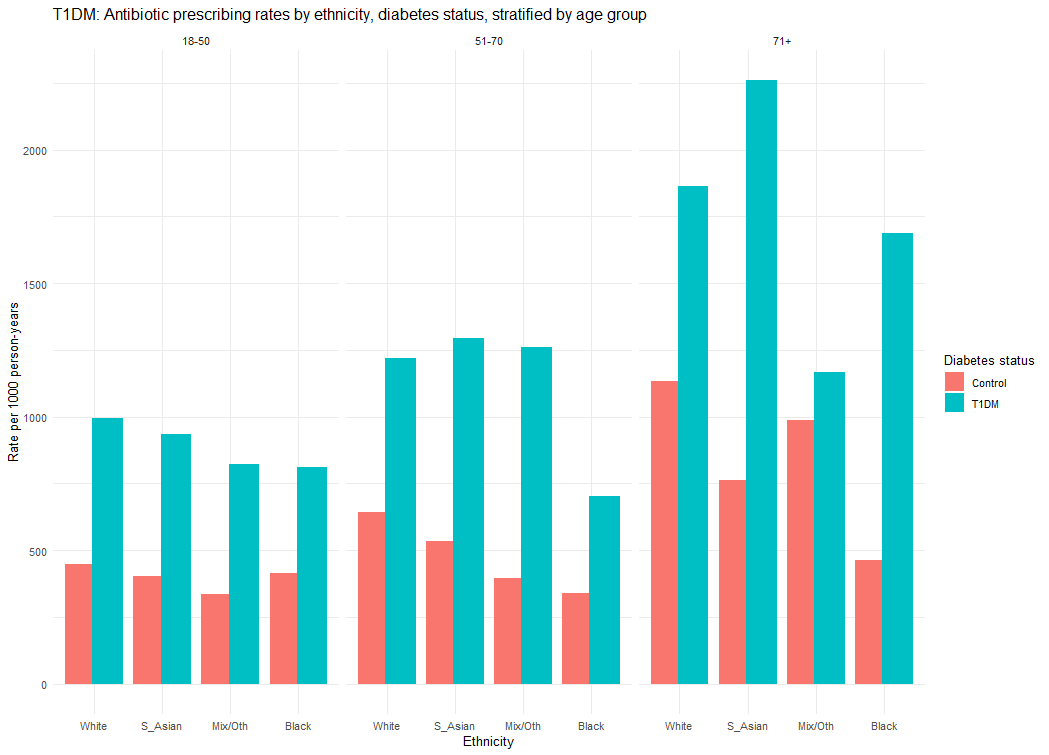


### **Supplementary Figure S5.** Antibiotic prescription rates for people with T2DM and matched controls, stratified by age group and ethnicity


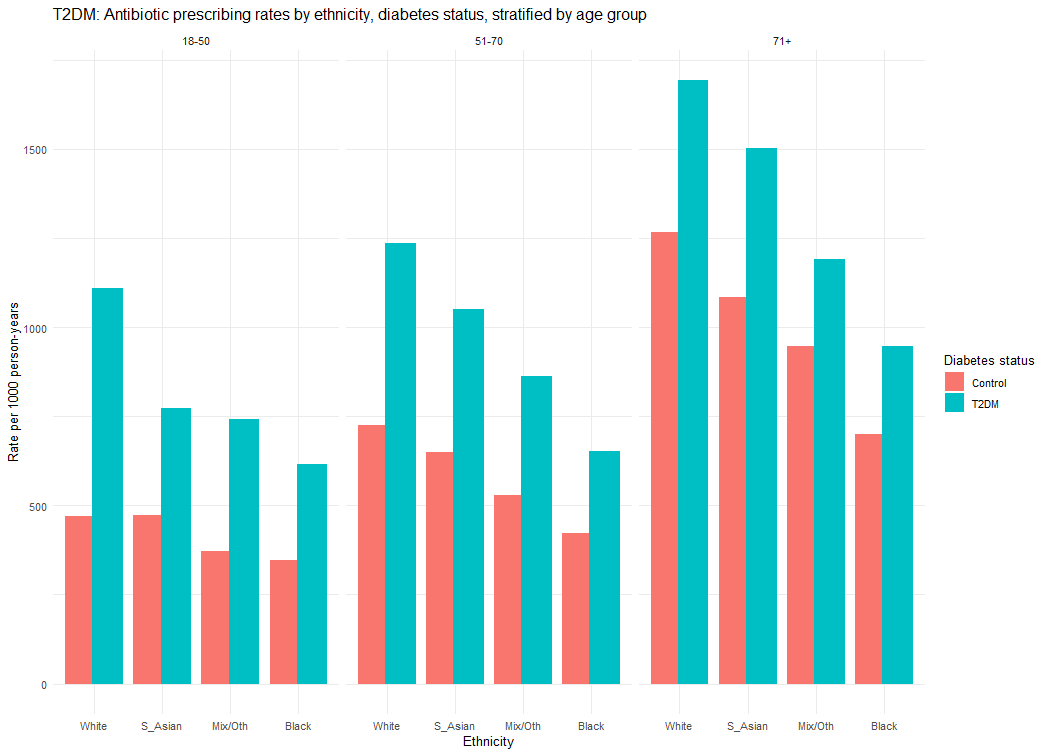


## **High prescription count analyses**

### **Supplementary Table S8.** Number and proportion of ‘high prescription’ individuals* in each diabetes group

|  | **T1DM** | | **T2DM** | |
| --- | --- | --- | --- | --- |
|  | **Cases** | **Controls** | **Cases** | **Controls** |
| **Total subjects** | 33,843 | 67,587 | 524,285 | 1,029,722 |
| **Total antibiotic courses** | 160,081 | 151,238 | 2,918,090 | 3,805,948 |
| **High prescription individuals** | 46 (0.14%) | 15 (0.02%) | 1135 (0.22%) | 945 (0.09%) |
| **Median (IQR) antibiotic courses per year** | 40.1 (32.7-51.9) | 37.6 (31.1-59.9) | 39.4 (30.0-50.5) | 37.4 (30.6-51.4) |
| **Proportion of total antibiotic courses given to high prescription individuals** | 7,593 (4.7%) | 2,782 (1.8%) | 161,626 (5.5%) | 130,183 (3.4%) |

*High prescription individuals were defined as individuals who had a prescription rate equivalent to fortnightly (or more frequently) during at least 6 months of follow-up time

### **Supplementary Figure S6.** Dominant antibiotic class among all ‘high prescription’ individuals

**
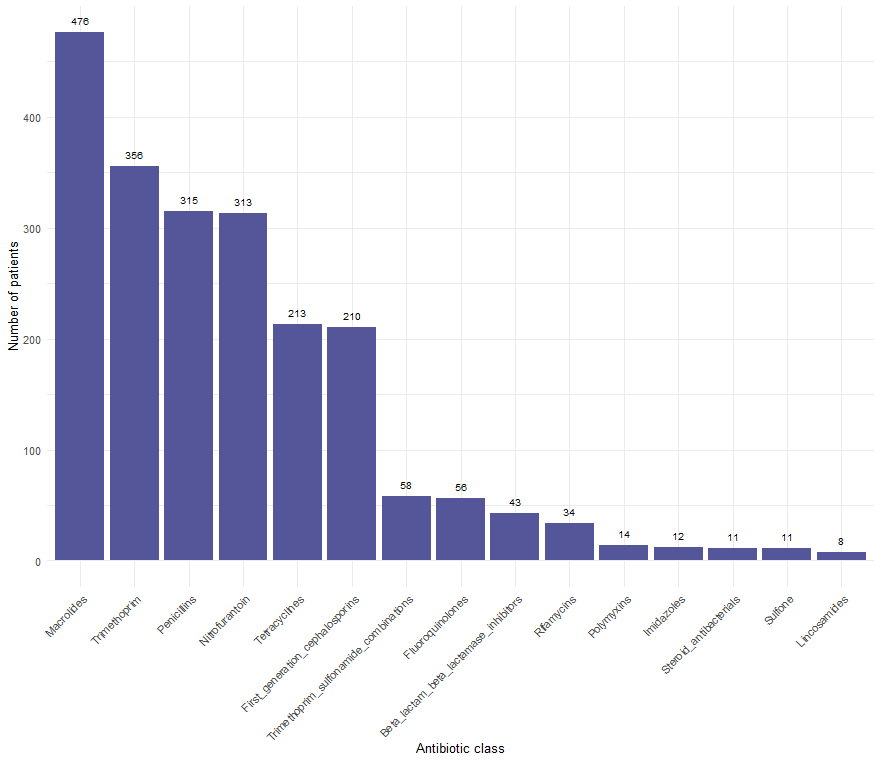
**

*The following classes were the dominant class for 5 or fewer patients: amphenicols, second generation cephalosporins, aminoglycosides, glycopeptides.

### **Supplementary Figure S7.** Dominant antibiotic class among high-prescription individuals in the T2DM cohort

**
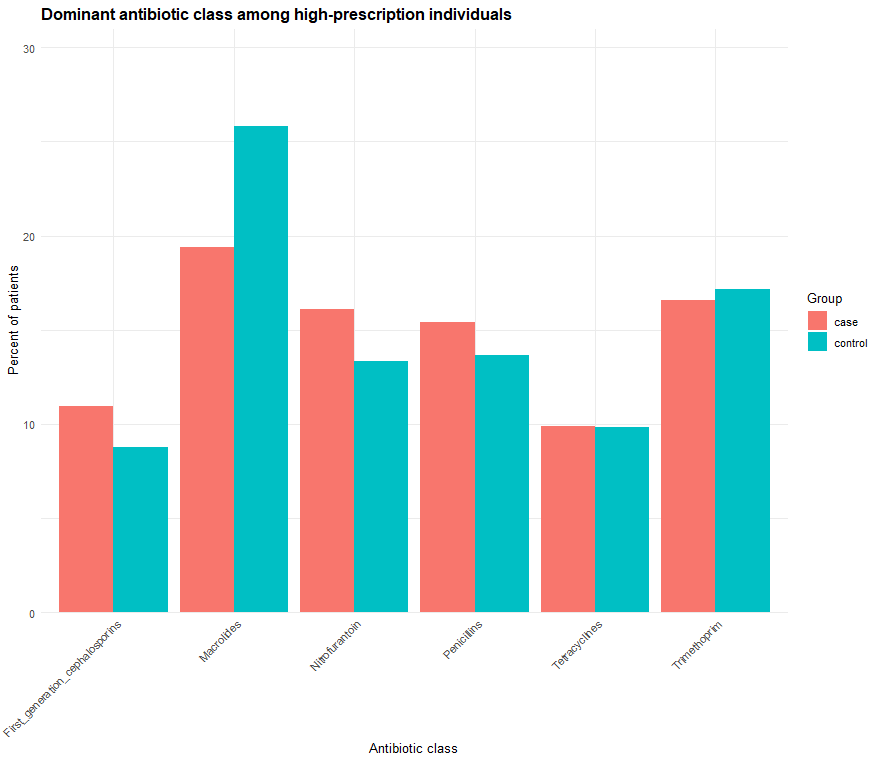
**

*****Note too few individuals with T1DM were identified as high-prescription individuals to enable breakdown by antibiotic class.

## **Acute infection antibiotic prescription analyses**

### **Supplementary Table S9.** Number of subjects with at least one UTI, cellulitis, or community acquired pneumonia (CAP) infection code during study follow up, and total UTI infection code episodes

|  | **T1DM** | | **T2DM** | |
| --- | --- | --- | --- | --- |
|  | **Cases** | **Controls** | **Cases** | **Controls** |
| **Total subjects** | 33,843 | 67,587 | 524,285 | 1,029,722 |
| **Subjects with ≥ 1 UTI infection code** | 2,079 | 2,309 | 45,399 | 60,964 |
| **Total episodes of coded UTI** | 3,698 | 3,705 | 84,152 | 107,721 |
| **Subjects with ≥ 1 cellulitis infection code** | 1,908 | 1,356 | 41,917 | 46,927 |
| **Total episodes of coded cellulitis** | 4,362 | 2,895 | 114,655 | 112,385 |
| **Subjects with ≥ 1 CAP infection code** | 4,221 | 4,950 | 91,703 | 131,156 |
| **Total episodes of coded CAP** | 7,896 | 8,593 | 184,726 | 253,031 |

### **Supplementary Table S10**. Combinations of community acquired pneumonia antibiotics prescribed on the same day following acute lower respiratory tract infection event. Results presented as number of instances of each combination per group, with percentage denoting the proportion each combination represents within each patient cohort.

| **Antibiotic combination** | **T1DM** | | **T2DM** | |
| --- | --- | --- | --- | --- |
|  | **Case** | **Control** | **Case** | **Control** |
| Amoxicillin + Clarithromycin | n=22 (52.3) | n=27 (65.9) | n=575 (62.2) | n=909 (64.1) |
| Amoxicillin + Clarithromycin + Doxycycline | NA | NA | NA | n=<5 |
| Amoxicillin + Doxycycline | n=10 (23.8) | n=8 (19.5) | n=227 (24.6) | n=293 (20.6) |
| Amoxicillin + Erythromycin | n=<5 | n=<5 | n=18 (1.9) | n=28 (2.0) |
| Clarithromycin + Doxycycline | n=8 (19.0) | n=<5 | n=88 (9.5) | n=159 (11.2) |
| Clarithromycin + Erythromycin | NA | NA | n=6 (0.6) | n=11 (0.8) |
| Doxycycline + Erythromycin | NA | n=<5 | n=10 (1.1) | n=16 (1.1) |

References

1. Lim WS, van der Eerden MM, Laing R, Boersma WG, Karalus N, Town GI, et al. Defining community acquired pneumonia severity on presentation to hospital: an international derivation and validation study. Thorax. 2003 May 01;58(5):377–82.
